# The Proinflammatory RAGE/NF-κB Pathway Is Involved in Neuronal Damage and Reactive Gliosis in a Model of Sleep Apnea by Intermittent Hypoxia

**DOI:** 10.1371/journal.pone.0107901

**Published:** 2014-09-29

**Authors:** Maria Florencia Angelo, Alejandra Aguirre, Rolando X. Avilés Reyes, Alejandro Villarreal, Jerónimo Lukin, Matías Melendez, Virginia Vanasco, Phil Barker, Silvia Alvarez, Alberto Epstein, Diana Jerusalinsky, Alberto Javier Ramos

**Affiliations:** 1 Instituto de Biología Celular y Neurociencia “Prof. E. De Robertis”, Facultad de Medicina, Universidad de Buenos Aires, Ciudad de Buenos Aires, Argentina; 2 Centre Internationale de Recherche en Infectiologie, INSERM U1111, CNRS UMR5038, Ecole Normale Supérieure, Université Lyon 1, Lyon, France; 3 Montreal Neurological Institute, McGill University, Montreal, Quebec, Canada; 4 Instituto de Bioquímica y Medicina Molecular, Facultad de Farmacia y Bioquímica, Universidad de Buenos Aires, Ciudad de Buenos Aires, Argentina; University of Nebraska Medical Center, United States of America

## Abstract

Sleep apnea (SA) causes long-lasting changes in neuronal circuitry, which persist even in patients successfully treated for the acute effects of the disease. Evidence obtained from the intermittent hypoxia (IH) experimental model of SA has shown neuronal death, impairment in learning and memory and reactive gliosis that may account for cognitive and structural alterations observed in human patients. However, little is known about the mechanism controlling these deleterious effects that may be useful as therapeutic targets in SA. The Receptor for Advanced Glycation End products (RAGE) and its downstream effector Nuclear Factor Kappa B (NF-κB) have been related to neuronal death and astroglial conversion to the pro-inflammatory neurodegenerative phenotype. RAGE expression and its ligand S100B were shown to be increased in experimental models of SA. We here used dissociated mixed hippocampal cell cultures and male Wistar rats exposed to IH cycles and observed that NF-κB is activated in glial cells and neurons after IH. To disclose the relative contribution of the S100B/RAGE/NF-κB pathway to neuronal damage and reactive gliosis after IH we performed sequential loss of function studies using RAGE or S100B neutralizing antibodies, a herpes simplex virus (HSV)-derived amplicon vector that induces the expression of RAGEΔcyto (dominant negative RAGE) and a chemical blocker of NF-κB. Our results show that NF-κB activation peaks 3 days after IH exposure, and that RAGE or NF-κB blockage during this critical period significantly improves neuronal survival and reduces reactive gliosis. Both *in vitro* and *in vivo*, S100B blockage altered reactive gliosis but did not have significant effects on neuronal survival. We conclude that both RAGE and downstream NF-κB signaling are centrally involved in the neuronal alterations found in SA models, and that blockage of these pathways is a tempting strategy for preventing neuronal degeneration and reactive gliosis in SA.

## Introduction

Sleep apnea (SA) is a highly prevalent pathology in adult humans. SA patients suffer a repeated and transient reduction in oxygen tension, termed intermittent hypoxia (IH). The central nervous system (CNS) is vulnerable to these hypoxic conditions, and neurocognitive manifestations of SA include not only daytime sleepiness, but also alterations in personality and impairment of concentration, perception, memory, communication and learning [1]–[5]. Continuous positive airway pressure therapy (CPAP) reduces daytime sleepiness and the cardiovascular complications of SA [6]. However, even in patients under CPAP, executive dysfunction often persists, possibly as a consequence of structural and functional alterations in brain neurocircuitry [7]–[9]. Furthermore, imaging studies have shown persistent structural alterations in the hippocampus of SA patients [10].

Regarding experimental settings, neuronal alterations and reactive gliosis have also been demonstrated in different paradigms that model the SA-induced IH [11], [12]. The cognitive impairments found in animal studies are structurally related to changes in hippocampal and cortical areas [13]–[18]. The precise mechanisms that lead to neuronal alterations are not fully understood, but production of reactive oxygen species (ROS) during the reoxygenation period (reviewed in [19]), glutamate-induced excitotoxicity [20] and inflammation [21], [22] have all been implicated in the development of the neuronal pathology.

Reactive astrogliosis is a general response of astrocytes to different types of injury; this reaction may reduce neuronal survival due to the secretion of pro-inflammatory cytokines, ROS and nitric oxide (NO). The subsequent formation of the glial scar also impedes neuronal reconnection (reviewed in [23], [24]). However, there are abundant data showing that reactive astrocytes are beneficial for the recovery of CNS function. For example, reactive astrocytes can produce energy substrates and trophic factors for neurons and oligodendrocytes, act as free radicals and glutamate scavengers, actively restore the blood-brain barrier, promote neovascularization, restore CNS ionic homeostasis, promote remyelination and stimulate neurogenesis from neural stem cells (reviewed in [25]–[27]). It is now known that reactive astrogliosis is a complex phenomenon leading to either pro-survival or pro-inflammatory neurodegenerative responses, involving the activation of different sets of genes [28]. Thus, an interesting target for facilitating neuroprotection in the injured brain would be to control the extent of the glial pro-inflammatory neurodegenerative response.

The cellular and molecular elements contributing to the inflammation are those involved in the activation of the innate immune response. The Receptor for Advanced Glycation End products (RAGE) is a Pattern Recognition Receptor (PRR) that participates in the innate immune response. Activation of this process can be achieved by interaction of PRR with Damage Associated Molecular Pattern (DAMP) proteins, and usually leads to pro-inflammatory responses mediated by the NF-κB transcription factor. RAGE activation by glial S100B, a DAMP released by astrocytes after injury, leads either to neuronal survival or death, depending on the level of NF-κB transcriptional activity [29] and activates astrocytes, ultimately promoting a pro-inflammatory response [30], [31].

In IH experimental models, RAGE is expressed in hippocampal and cortical areas related to the cognitive disorders expressed in SA patients [11], [22]. S100B levels have also been found increased in SA patients [32], [33]. In an attempt to understand the participation of RAGE signaling and NF-κB role on neuronal survival and reactive gliosis after IH, we here report the results of loss of function studies *in vitro* and *in vivo* performed by blocking different steps of the S100B/RAGE/NF-κB pathway. By using RAGE blocking antibodies, chemical blockers of NF-κB activation and by developing a HSV-derived amplicon vector that induces the expression of a defective RAGE (RAGEΔcyto), we have been able to show that attenuation of the RAGE/NF-κB signaling leads to an improved neuronal survival and to a reduced reactive gliosis after IH exposure.

## Materials and Methods

### Materials

Antibodies were obtained from Sigma (mouse monoclonal anti-S100B); Dako [rabbit polyclonal anti gliofibrillary acidic protein (GFAP)]; Millipore [mouse monoclonal anti microtubule associated protein (MAP-2); mouse monoclonal anti-neuronal nuclei (NeuN), mouse monoclonal anti-RAGE, mouse monoclonal anti-GFP, mouse monoclonal anti-nuclear localization signal of p65 NF-κB subunit (p65NLS), rabbit polyclonal anti-p65 NF-κB subunit]; Pierce (mouse monoclonal anti βIII-Tubulin) and ICN Biomedicals (rabbit polyclonal anti-β-galactosidase). Secondary biotinylated antibodies, streptavidin complex (Extravidin) used for immunohistochemistry studies, 4-chloro-5-bromo-3-indolyl-β-galactoside (X-gal) and other chemicals were purchased from Sigma. Secondary fluorescent antibodies were obtained from Jackson ImmunoResearch (Baltimore Pike, West Groove, PA). All other chemical substances were of analytical grade.

### Animals

Adult male Wistar rats (200–250 g) and 3-day old rat pups obtained from the animal facility of the School of Pharmacy and Biochemistry (University of Buenos Aires) and adult male transgenic mice expressing an NF-κB-LacZ reporter gene (30 g) [34] from the Montreal Neurological Institute and Hospital (Center for Neuronal Survival, McGill University) were used in this study. Animals were housed in a controlled environment (12/12-h light/dark cycle, controlled humidity and temperature, free access to standard laboratory rat food and water). Animal care and all procedures done for this experimental protocol were in accordance with the NIH guidelines for the Care and Use of Laboratory Animals, and the principles presented in the Guidelines for the Use of Animals in Neuroscience Research by the Society for Neuroscience. Protocols were approved by the CICUAL Animal Committee of the School of Medicine, University of Buenos Aires.

### Dissociated mixed hippocampal cell cultures

This procedure was performed according to Lee and Parpura [35] with minor modifications. Hippocampi were obtained after brain dissection of deeply anaesthetized 3-day old Wistar rats and incubated for 1 h with papain (20 U/ml) at 37°C in 5% CO_2_. Papain was removed and tissue was washed once in DMEM. Hippocampi were mechanically dissociated with a fire-polished glass serological pipette until no visible clamps remained. Cells were plated onto poly-L-lysine-coated multiwell chambers and fed with DMEM, 1% glutamine, 1% penicillin-streptomycin and 10% fetal calf serum. Cultures were maintained at 37°C in a humidified atmosphere with 5% CO_2_; 50% of the medium was replaced by fresh medium every 3 days. This protocol was kindly provided by Dr. Vladimir Parpura (UAB, USA). All experiments were performed in cultured cells after for 9-11 days.

### Exposure to Intermittent Hypoxia

The *in vivo* IH experiments were performed as described in Aviles Reyes et al. [11]. Briefly, animals were randomly divided into four experimental groups and placed into two identical plastic normobaric chambers (8 L). During the light period, O_2_ was reduced from 21% to 10% over 1 min, held at 10% for 5 min, returned to 21% over 1 min, and held at 21% for 6 min. This cycle was repeated continuously for 8 h, giving a minimum of five hypoxic events per hour of sleep, in accordance with the clinical definition of sleep apnea [6]. Groups exposed to IH were named IH1, IH3, IH5 and IH10, representing animals that have undergone exposure to IH for 1, 3, 5 and 10 days. Control animals were housed for the same period in identical chambers where the only source of gas was room air. O_2_ level in each chamber was monitored continuously with an electrochemical sensor connected to a digital oxymeter (PumpControl, Buenos Aires, Argentina) and regulated by timer-controlled valves connected to room air and to a N_2_ source equipped with separated flow mixers.

The *in vitro* IH exposure was performed as described in Shan et al. [36]. Briefly, dissociated mixed hippocampal cell cultures were exposed to IH (cycles of normoxia: 21% O_2_, 5% CO_2_, balance N_2_ for 25 min; and hypoxia: 0.1% O_2_, 5% CO_2_, balance N_2_ for 35 min). Control cultures were kept under normoxia conditions (21% O_2_, 5% CO_2_, balance N_2_). For the blocking assays, final concentrations of the blocking agents were: anti-RAGE 1 µg/ml, anti-S100B 1 µg/ml, BAY117082 2 µM, mouse IgG control antibody 1 µg/ml, DMSO 0.02% (v/v) (vehicle for BAY117082).

### Surgery and Infusion Procedures

Rats were anaesthetized with ketamine/xylazine (90/10 mg/kg ip) and 22G guide cannulae were implanted in the CA1 region of the dorsal right hippocampus, at stereotaxic coordinates A−4.3, L-3.0, V−1.4 of the atlas by Paxinos and Watson [37], following the protocol described by Slipczuk et al. [38]. The cannulae were fixed to the skull and immobilized with dental acrylic. Animals were allowed to recover from surgery for three days. Cannulated rats received daily infusions of 1 µl 15 min before IH exposure. The neutralizing anti-RAGE and anti-S100B antibodies or control IgG were diluted to working concentration (0.5 µg/µL) with sterile PBS. The NF-κB blocker sulfasalazine was diluted to a working solution (1.25 mM) with sterile PBS from a DMSO-based stock solution. Infection with the HSV-derived amplicon was achieved by a unique intra-hippocampal injection of the defective virus expressing the desired construct. Animals were exposed to IH two days later to allow maximal construct expression.

### Fixation

Animals were deeply anaesthetized with ketamine/xylazine (90/10 mg/kg, ip) and were perfused through the left ventricle, initially with saline solution containing 5000 UI of heparin and subsequently with a fixative solution containing 4% w/v paraformaldehyde in 0.1 M phosphate buffer, pH 7.2. Following delivery of 300 mL of fixative solution through a peristaltic pump, brains were removed and kept in cold fixative solution for 2 h. Brains were then washed three times in cold 0.1 M phosphate buffer pH 7.4 containing 5% (w/v) sucrose, left in washing solution for 18 h at 4°C and then washed in 0.1 M phosphate buffer pH 7.4 containing 30% w/v sucrose as cryoprotective. Then, brains were rapidly frozen at −80°C for 3 h and stored at −20°C. Coronal 50-µm-thick brain sections were cut using a cryostat. Sections were cryoprotected by immersion in a solution containing 30% (v/v) ethylene glycol and 20% (v/v) glycerol in 0.1 M phosphate buffer pH 7.4 at −20°C. Cultured cells were fixed as previously described in Villarreal et al [29]. Briefly, cells were washed with cold phosphate-buffered saline (PBS) and fixed with 4% paraformaldehyde plus 4% sucrose in PBS pH 7.2 for 15 min at 18–25°C.

### Immunohistochemistry and immunofluorescence

Brain sections of animals from all experimental groups were simultaneously processed in the free floating state as previously described [11]. All antibodies were diluted in a solution with phosphate-buffered saline (PBS), 1% Triton X-100 and 3% normal goat serum. Development of peroxidase activity was carried out with 0.035% w/v 3,3′ diaminobenzidine plus 2.5% w/v nickel ammonium sulfate and 0.1% v/v H_2_O_2_ dissolved in acetate buffer 0.1 M pH 6.0. Controls for the immunohistochemistry procedure were routinely performed by omitting the primary antibody; control sections did not develop any immunohistochemical labeling. Double fluorescent immunostaining studies were performed essentially in the same way, but the endogenous peroxidase inhibition was omitted and isotypic specific secondary antibodies (Jackson ImmunoResearch, West Grove, PA, USA) labeled with FITC or Rhodamine RRX were used in a 1∶800 dilution. Nuclear counterstaining was done with Hoechst 33342 (2 µg/ml). For immunocytochemistry, fixed cell cultures were washed three times with cold PBS and permeabilized with 0.1% Triton X-100. The procedure was then followed as stated for tissue sections using the indicated dilutions of the primary antibodies: GFAP 1∶5000, βIII-Tubulin 1∶5000, p65NLS 1∶1000. Digital photographs were taken in an Olympus IX-81 microscope equipped with a DP71 camera (Olympus, Tokyo, Japan) or in a Zeiss Axiophot (Carl Zeiss, Oberkochen, Germany) microscope equipped with a digital camera (Olympus Q5). Confocal images were taken in an Olympus FV-1000 confocal microscope.

### Thiobarbituric Acid Reactive Substances (TBARS) assay

Immediately after IH exposure, rats were deeply anaesthetized and sacrificed by decapitation. Hippocampi were dissected and homogenized in cold buffer containing K_2_HPO_4_/KH_2_PO_4_ 30 mM, KCl 120 mM, pH 7.4 and 10% (v/v) butylated hydroxytoluene (BHT) 4% in ethanol was added as antioxidant. Primary cell cultures were lysed within the same buffer immediately after finishing IH exposure. A 100 µl aliquot of hippocampal or cell homogenate was added to 200 µl of 0.1 N HCl, 30 µl 10% (w/v) phosphotungstic acid and 100 µl of 0.7% (w/v) 2-thiobarbituric acid. The mixture was heated in boiling water for 60 min. TBARS were extracted in 1 mL of n-butanol. After a brief centrifugation, the fluorescence of the butanolic layer was measured in a Hitachi F-3010 spectrophotometer at 515 nm (excitation) and 553 nm (emission). A calibration curve was prepared using 1,1,3,3-tetramethoxypropane as standard. Results were expressed as pmol of TBARS per mg of protein and normalized to the control TBARS level [39].

### RT-PCR

RT-PCR was performed as previously described in Ramos et al [40]. Brains from IH exposed animals were dissected, hippocampal tissue extracted and mRNA was isolated using the RNAeasy Mini kit, according to the manufacturer's instructions (Qiagen, Hilden, Germany). cDNA was generated using the Omniscript RT kit (Qiagen) with random hexamers (Roche Products), and PCR was performed using specific primers for IκBα (forward 5′-GCAATCATCCACGAAGAGAAGCC-3′, reverse 5′-TTACCCTGTTGACATCAGCCCC -3′), XIAP (forward 5′-TGGTCAGAACACAGGAGACACTTTC-3′, reverse 5′- CACTTCACTTTATCGCCTTCACC-3′) or BclXL (forward 5′- AGTAAACTGG GGTCGCATCGTG-3′, reverse 5′- GTAGTGGTTCTCCTGGTAGCAATGG-3′). Detailed PCR protocols are available from authors under request. PCR products were run in a 1.5% agarose gel and photographed in a Bio-Rad (Hercules, CA) VersaDoc 4000 imaging system. Each RT-PCR experiment was run with negative controls, in which Omniscript reactions were performed in the absence of reverse transcriptase; the negative controls consistently failed to generate a PCR product. Quantitative analysis of gel images was done with the ImageJ software.

### *β*-Galactosidase assays

Fixed brain sections were assayed for β-galactosidase activity as previously described in Bhakar et al. [34]. Briefly, sections were incubated at 37°C in 80 mM dibasic sodium phosphate, 20 mM monobasic sodium phosphate, 2 mM MgCl_2_, 0.2% Nonidet P-40, 1 mg/ml sodium deoxycholate, 5 mM potassium ferricyanide, 5 mM potassium ferrocyanide, and 800 µg/ml 4-chloro-5-bromo-3-indolyl-β-galactoside for 4–16 hr. Samples were then washed in PBS and post-fixed in 4% paraformaldehyde in PBS.

### Viral vector production

#### Amplicon plasmids

Plasmids were constructed carrying either RAGEwt, RAGE-Δcyto or pcDNA3 backbone (control). The pcDNA3-RAGEwt and pcDNA3-RAGEΔcyto plasmids (kindly provided by H. Huttunen, Neuroscience Center, University of Helsinski) were digested with KpnI, blunt-ended and digested with XbaI. The RAGE containing sequences were cloned into the *Nhe*I-blunt and XbaI ended sites of amplicon plasmid pA-EUA2 [41] to generate the RAGE-expressing amplicon plasmids. It is noteworthy that all amplicon plasmids also express GFP reporter gene from an independent transcription unit.

#### Amplicon vectors

Amplicon vector stocks were prepared as already described, using the amplicon plasmids, and the highly neuroattenuated HSV-1LaLΔJ virus as helper [41], [42]. Briefly, 7b cells, which are Vero cells expressing HSV-1 ICP4 and ICP27 proteins [43], were independently transfected with 5 µg of each amplicon plasmid using Lipofectamine Plus (Invitrogen). One day later, transfected cells were superinfected at a multiplicity of infection of 0.3 plaque forming unit (PFU) per cell, with HSV-1LaLΔJ as helper virus. When cytopathic effect was maximal, cells were collected by centrifugation, disrupted by three freeze/thaw cycles to release vector stocks, and re-centrifuged at 1000 g for 10 min to pellet the cell debris. Helper and vector particles in the supernatants were then titrated as already described [41], [44]. Cells expressing fluorescent GFP were scored directly under an inverted fluorescence microscope (Olympus, Tokyo, Japan). Titers of the different amplicon vector stocks ranged from 1×10^6^ to 4×10^7^ transducing units (TU) per ml.

### Morphometric analysis

In order to ensure objectivity all measurements were performed on coded slices. GFAP immunostained area and feature assessment of astroglial cells, morphometric parameters of βIII-Tubulin stained neurites, features of NeuN stained neuronal cells counts and p65NLS stained cells were performed using the NIH ImageJ software. For immunohistochemistry, images taken with the microscope were captured with the digital camera, transformed to 8-bits gray scale, normalized and an interactive threshold selection was carried out. Once the threshold was determined it was kept fixed for the entire experiment. For the analysis of neuronal alterations and NeuN staining, counting was done interactively discriminating the type of labeling observed in the neuronal nuclei. Quantification of NeuN+ neurons was performed by dividing the population into two categories: normal staining (intense NeuN+ nucleus plus light cytoplasm) and abnormal staining (spongiform nuclear NeuN+ staining or cells showing NeuN+ cytoplasmic staining with nuclear NeuN negligible staining) as previously described [11] In all cases (neuronal or glial markers), approximately 10–15 fields per tissue section per treatment per anatomical area (hippocampus, cortex) and marker were analyzed. Sections coming from six to eight animals per treatment were analyzed. For the *in vitro* morphology studies, glial cells were divided into three populations: Filamentous astrocytes (with long prolongations and small perinuclear soma), polygonal astrocytes (big soma without prolongations) and intermediate astrocytes (combination of both morphologies). For length quantification of the beta-3-tubulin immunoreactive neuronal projections, the NeuronJ plug-in of the ImageJ software (National Institutes of Health) was used. The mean total neurite length per neuron was calculated for each microscopic field and referred to the control values to render a relative measurement.

### Statistical analysis

Experiments and measurement were done 3–4 times showing identical results. Data were normalized and presented as pooled data in the graphs. Statistical comparisons were analyzed with one-way ANOVA and Student-Newman-Keuls post-test, or two-way ANOVA and Bonferroni post-test (as described in each case) using GraphPad Software (GraphPad Software Inc., San Diego, CA, USA).

## Results

### In vitro IH exposure parallels the increase in ROS, neuronal alterations and reactive gliosis observed in vivo

**Figure 1:**
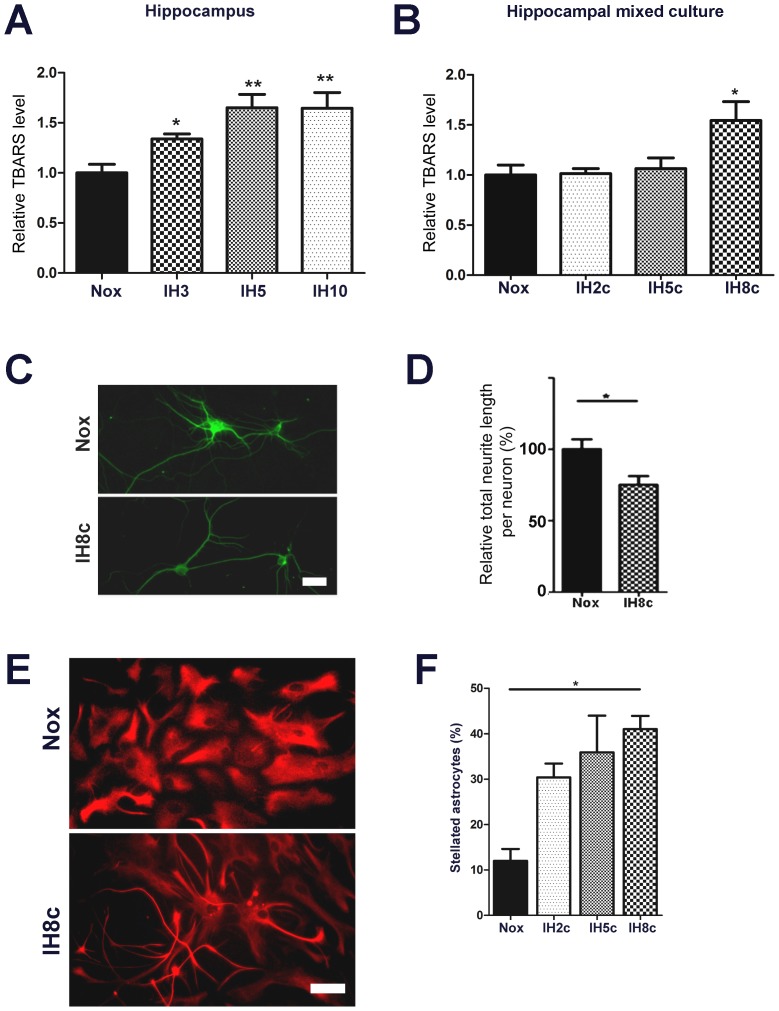
IH-exposed dissociated mixed hippocampal cell culture reproduces several features of in vivo IH exposure. A: Relative TBARS content in the hippocampus of animals exposed to normoxia (Nox) or to IH for 3, 5 or 10 days (IH3, IH5, IH10). B: Relative TBARS content in the dissociated mixed hippocampal cell culture exposed to normoxia (Nox) or to 2, 5, or 8 cycles of IH (IH2c, IH5c, IH8c). C: Beta-3-tubulin immunostained hippocampal neurons in the dissociated mixed hippocampal culture exposed to normoxia (Nox) or 8 cycles of IH (IH8c), bar  = 20 µm. D: Quantitative analysis of the relative neurite length in the hippocampal neurons in the mixed culture exposed to normoxia (Nox) or 8 cycles of IH (IH8c). E: GFAP immunostained astrocytes in the dissociated mixed hippocampal culture exposed to normoxia (Nox) or 8 cycles of IH (IH8c), bar  = 23 µm. F: Quantitative analysis of astroglial stellation followed by the phenotypic change in the GFAP-immunoreactive astrocytes after 2, 5, or 8 cycles of IH (IH2c, IH5c, IH8c). Data on the graphs are shown as means ±SEM; significance vs. control group was represented as indicated: * p<0.05; **p<0.01; *** p<0.001 after one way ANOVA and Student Newman Keuls post-test.

Experimental models of SA using the IH paradigm have demonstrated that increased oxidative stress is a crucial component of the SA pathophysiology, and that antioxidants are useful to prevent neuronal alterations [22], [36]. In the experimental paradigm of *in vivo* IH exposure, which reproduces the oxygen saturation in haemoglobin levels found in patients [11], we observed that TBARS level is significantly increased after 3 days of IH exposure (Figure 1A). When this parameter was studied in dissociated mixed hippocampal cell cultures, we found that 8 cycles of IH exposure were able to reproduce the magnitude of the TBARS increase observed *in vivo* (Figure 1B).

Animals exposed to IH have shown signs of neurodegeneration, as evidenced by relocalization of NeuN neuronal nucleus marker and shorter neuronal projections, as well as by a significant reactive gliosis [11]. Exposure of dissociated mixed hippocampal cell cultures to 8 cycles of IH induced a similar reduction in neurite extension (Figure 1C, D). IH also induced a reduction in the number of polygonal astrocytes, together with an increase in the abundance of cells in the fibrillar phenotype, a phenomenon known as stellation and considered to be the *in vitro* correlation of reactive gliosis (Figure 1E, F).

### IH exposure activates the NF-κB pathway

**Figure 2:**
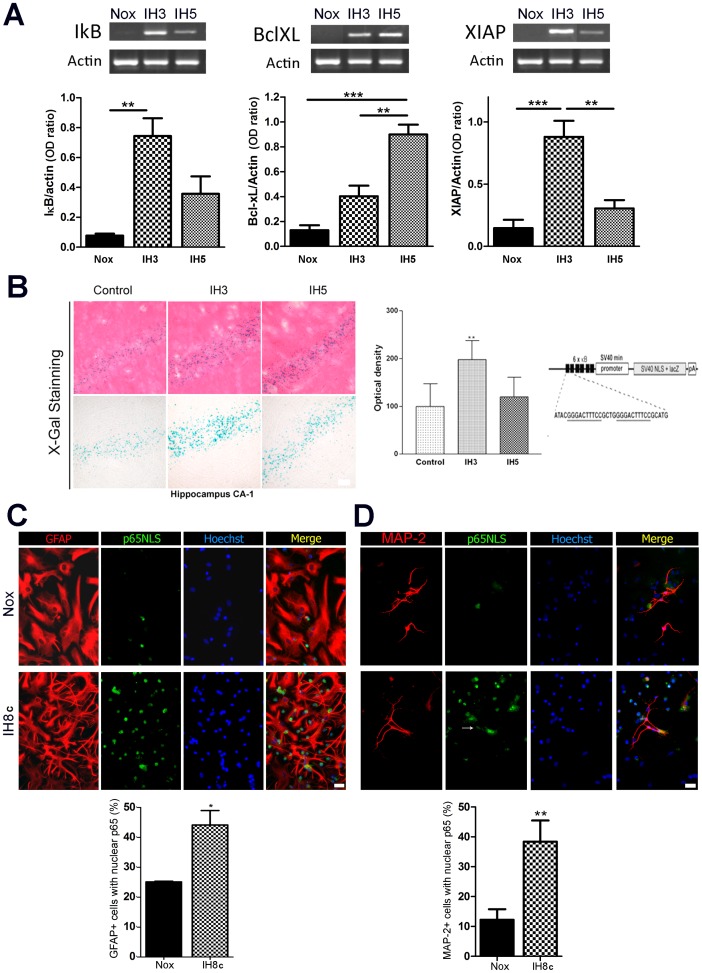
IH exposure activates NF-κB *in vitro* and *in vivo*. A: Animals were exposed to 3 or 5 days of IH and mRNA was isolated from hippocampal tissue, reverse transcribed and subjected to PCR using primers directed against IκB, Bcl-XL, XIAP and actin. Optical density of the bands was quantified with the ImageJ gel analyzer and related to the intensity of the actin bands. B: NF-κB reporter mice expressing an NF-κB reporter minigene showed increased NF-κB activity after 3 days of exposure to IH (IH3) in the hippocampal CA-1 region (left); quantitative evaluation of X-gal staining (right). NF-κB reporter minigene contains three tandem HIV-derived κB binding element repeats, placed proximal to a minimal promoter derived from SV40, an E. coli β-galactosidase cDNA with a mammalial Kozak consensus, an SV40 T-antigen-derived nuclear localization signal, and a polyA tract [34]. C: Immunocytochemistry for the p65 nuclear localization signal (p65NLS) in astrocytes of dissociated mixed hipocampal cell cultures, bar  = 20 µm (left), quantitative analysis (right) shows the percentage of GFAP+ cultured cells showing p65NLS nuclear staining after 8 cycles of IH exposure (IH8c) or normoxia (Nox). D: Immunocytochemistry for the p65 nuclear localization signal (p65NLS) in neurons of the dissociated mixed hipocampal cell culture, bar  = 20 µm (left), quantitative analysis (right) shows the percentage of MAP2+ cells showing p65NLS nuclear staining after 8 cycles of IH exposure (IH8c) or normoxia (Nox). Data on the graphs are shown as means ±SEM; significance vs. control group was represented as indicated: * p<0.05; **p<0.01; *** p<0.001 after one way ANOVA and Student Newman Keuls post-test.

We have previously shown increased RAGE expression and overexpression of the RAGE ligand S100B in animals exposed to IH [11]. Since RAGE canonically activates NF-κB mediated downstream signalling, our next question was whether NF-κB was activated after IH exposure. For that purpose animals were exposed to the IH paradigm for 3 or 5 days and then RT-PCR assays for several NF-κB target genes were performed in samples obtained from hippocampal tissue. As shown in Figure 2A, IκB, Bcl-XL and XIAP, all of them NF-κB target genes, were increased after IH exposure. To verify if the increase in these target mRNAs correlates with increased NF-κB transcriptional activity, transgenic mice expressing an NF-κB-LacZ reporter gene were exposed to IH, and LacZ expression was detected in tissue sections by histochemistry. As shown in Figure 2B, hippocampal NF-κB -induced transcriptional activity was significantly increased in IH exposed animals and presented a peak at IH3. In agreement with these results, astrocytes and neurons from the hippocampal mixed cultures also showed increased nuclear p65NLS immunoreactivity after IH exposure (Figure 2C, 2D), thus indicating activation of the NF-κB signalling.

### S100B blockage reduces reactive gliosis after IH exposure

S100B has been recently recognized as a DAMP released by astrocytes after acute or chronic brain injury, and it is a known RAGE ligand. S100B levels are increased in SA patients [32], [33] and its expression is induced in experimental models of SA [11], [12]. To study S100B role in the neuronal and glial alterations observed after IH, we blocked S100B using neutralizing antibodies in dissociated mixed hippocampal cell cultures and in animals exposed to IH.

**Figure 3:**
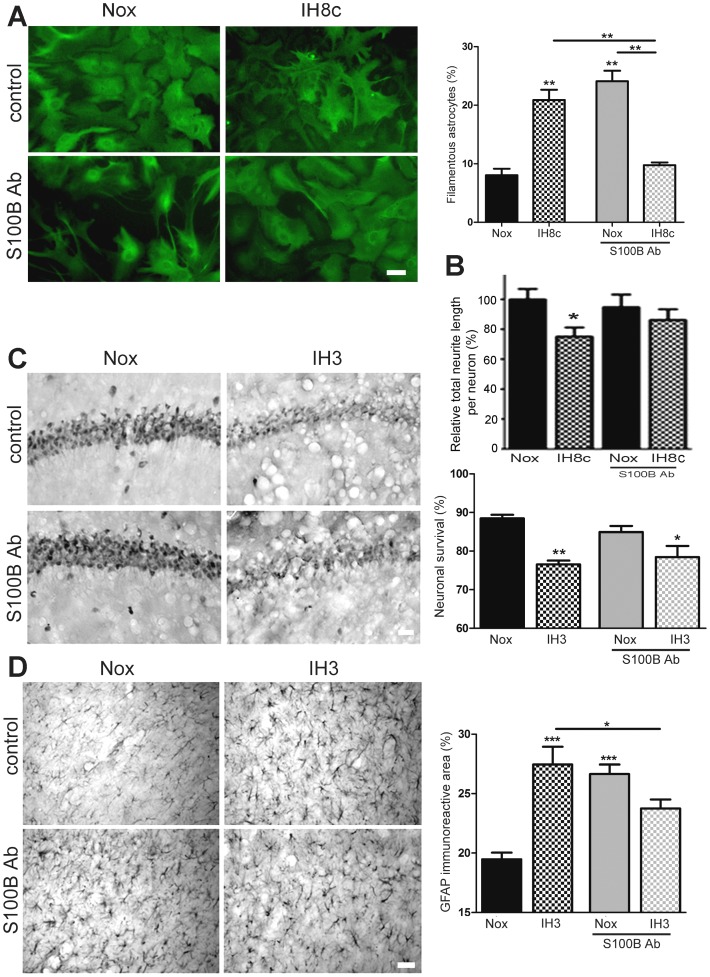
S100B blockage reduced astroglial stellation in vitro and reactive gliosis in vivo. A: GFAP immunostained astrocytes in dissociated mixed hippocampal cell cultures after 8 cycles of IH exposure (IH8c) in the absence or presence of S100B blocking antibodies (1 µg/ml), bar  = 23 µm (left). Percentage of filamentous astrocytes was evaluated in the same cultures using the ImageJ cell counter plugin and referred to the total number of GFAP+ cells (right). B: Relative neurite length per neuron in the dissociated mixed hippocampal cell culture after 8 cycles of IH exposure (IH8c) in the absence or presence of S100B blocking antibodies. C: NeuN immunostaining of hippocampal CA-1 region in cannulated animals that received S100B neutralizing antibodies and were exposed to three days of IH cycles (IH3), bar  = 40 µm (left). Quantitative analysis of the percentage of neuronal survival (i.e. those neurons showing normal NeuN staining) in sections obtained from these animals (right). D: GFAP immunostaining of astrocytes in the hippocampal CA-1 region in cannulated animals that received S100B neutralizing antibodies and were exposed to three days of IH cycles (IH3) or normoxia (Nox), bar  = 40 µm (left). Quantitative analysis of the percentage of the area covered by GFAP-immunoreactive cells (right) is used to evidence increased area occupied by hypertrophied reactive astrocytes. Data on the graphs are shown as means ±SEM; significance vs. control group was represented as indicated: * p<0.05; **p<0.01; *** p<0.001 after two-way ANOVA and Bonferroni post-test.

As shown in figure 3A, S100B blockage abolished the astrocytic stellation induced by 8 cycles of IH in mixed hippocampal cell culture but significantly increased stellation in normoxic conditions. S100B neutralizing antibodies failed to significantly prevent the reduction of neurite length induced by IH *in vitro* (Figure 3B). In agreement with this result, S100B neutralizing antibodies administrated intra-hippocampally were unable to prevent the neuronal degeneration induced by 3 days of exposure to IH in animals (Figure 3C). This S100B blockage was, however, able to partially reduce astroglial hypertrophy in animals exposed to IH (Figure 3D). Similarly, as it was previously shown *in vitro*, S100B blockage in normoxic conditions induced reactive gliosis in animals from the normoxic group (Figure 3D).

### RAGE blockage reduces neuronal alterations and reactive gliosis after IH exposure

RAGE is a pattern recognition receptor that can be activated by DAMP proteins released after brain injury, including glial S100B. RAGE engagement by its ligands leads to increased NF-κB activity and innate immunity activation. In order to study whether RAGE activity is required for the neuronal and glial alterations observed after IH exposure, two different approaches were implemented for the loss of function studies; RAGE blocking antibodies were used to neutralize endogenous RAGE and a HSV-derived amplicon vector expressing a dominant negative RAGE (RAGEΔcyto) unable to bind intracellular adaptor proteins was used to compete by sequestering endogenous RAGE ligands. For the gain of function studies, an HSV-derived amplicon bearing a full length RAGE (RAGEwt), was used.

**Figure 4:**
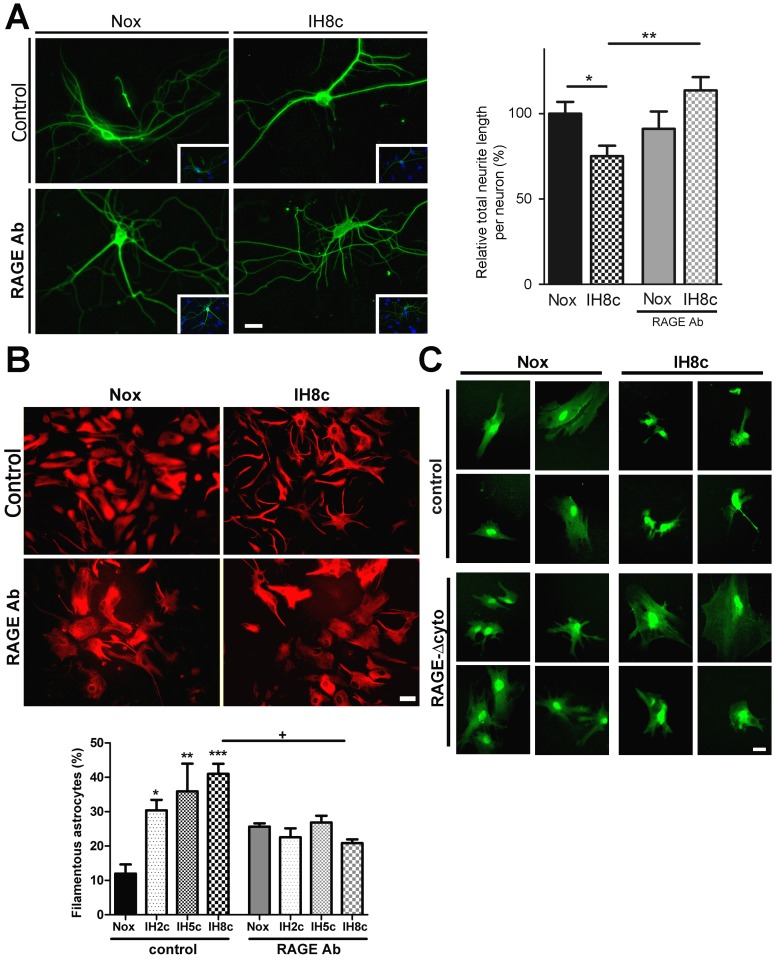
RAGE is required for the neuronal alterations and astroglial stellation induced by IH exposure in the dissociated mixed hippocampal cell culture. A: βIII-Tubulin immunostained hippocampal neurons in dissociated mixed hippocampal cell culture after 8 cycles of IH exposure (IH8c) or normoxia (Nox) in the absence and presence of RAGE blocking antibodies (1 µg/ml), bar  = 14 µm (left). Quantitative analysis of relative neurite length per neuron in a similar experiment (right). B: GFAP immunostained astrocytes in dissociated mixed hippocampal cell culture after 8 cycles of IH exposure (IH8c) or normoxia (Nox) in the absence or presence of RAGE blocking antibodies, bar  = 14 µm (top). Quantitative analysis of the percentage of filamentous astrocytes (bottom). C: Astrocytes infected with the HSV-derived amplicon driving the expression of RAGEΔcyto and GFP, or control amplicon expressing only GFP, exposed to 8 cycles of IH exposure (IH8c) or normoxia (Nox), bar  = 12 µm. Data on the graphs are shown as means ±SEM; significance vs. control group was represented as indicated: * p<0.05; **p<0.01; *** p<0.001 after two way ANOVA and Bonferroni post-test.

As shown above, exposure to IH significantly reduced neurite length in hippocampal cultured neurons from mixed culture. This detrimental effect was prevented by incubation with anti-RAGE neutralizing antibodies (Figure 4A). Similarly, IH exposure induced astrocyte stellation, and RAGE blockage efficiently prevented this effect as well (Figure 4B). Astrocytes infected with the HSV-derived amplicon expressing RAGEΔcyto, but not with the amplicon bearing the control construct, were also resistant to IH-induced stellation (Figure 4C).

**Figure 5:**
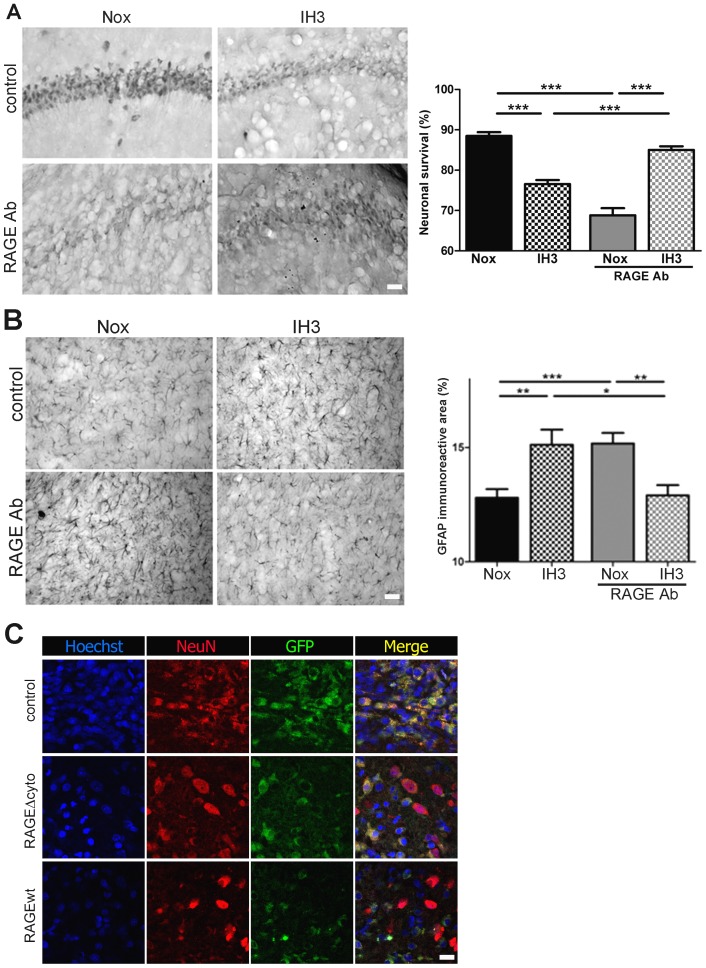
RAGE is required for the neuronal alterations and reactive gliosis induced by IH *in vivo*. NeuN (A) or GFAP (B) immunostaining in the hippocampal CA-1 region of animals exposed to IH for three days (IH3) and treated with intrahippocampal administrations of RAGE blocking antibodies or control IgG, bar  = 40 µm. Graphs on the right show the quantitative analysis of the percentage of neuronal survival and of the area covered by GFAP-immunoreactive cells. C: Confocal images of animals that were infected with the HSV-derived amplicon driving the expression of RAGE (RAGEwt), RAGEΔcyto or empty sequence and were exposed to IH for three days, bar  = 15 µm. Data on the graphs are shown as means ±SEM; significance vs. control group was represented as indicated: * p<0.05; **p<0.01; *** p<0.001 after two way ANOVA and Bonferroni post-test.

We then analyzed if the RAGE blockage was also able to reduce neuronal alterations and reactive gliosis induced by IH exposure *in vivo*. For that purpose, animals were cannulated unilaterally and RAGE blocking antibodies, or the unrelated control IgG, were infused before exposing animals to the IH cycles. This procedure was repeated every day, 15 min before initiating IH exposure. In agreement with our previous results, IH exposure reduced neuronal survival, an effect that was evidenced by atypical localization of NeuN and shorter neurite projections on animals that received control antibodies (Figure 5A and [11]). The infusion of RAGE blocking antibodies reduced the number of abnormal neuronal nuclei (i.e. those showing absence of NeuN staining in the nucleus and relocalization to the cytoplasm) in animals exposed to IH, but surprisingly, RAGE blockage was detrimental in normoxic conditions (Figure 5A). On the other hand, IH exposure also induced a profuse reactive gliosis as previously described [11] and infusion of RAGE blocking antibodies efficiently prevented reactive gliosis induced by IH, but caused reactive gliosis *per se* in normoxic conditions (Figure 5B). Infection with the amplicon bearing the sequence of RAGEΔcyto also reduced the neuronal loss induced by IH exposure, while RAGEwt overexpression increased neuronal alterations in IH exposed animals (Figure 5C).

### Inhibition of NF-κB transcriptional activity improves neuronal survival and reduces reactive gliosis after IH exposure

NF-κB transcriptional activity response was reported to be increased in the periphery in SA patients [45]–[47]. The generic downstream response after RAGE engagement with its ligands is a higher NF-κB transcriptional activity. Since we have shown that increased RAGE expression and activity render in reactive gliosis and neuronal alterations after IH, our next question was whether NF-κB was required for those effects. For that purpose, and to further dissect the molecular pathways involved in the observed neuroglial effects, we blocked NF-κB activity *in vitro* and *in vivo* after exposure to IH.

**Figure 6:**
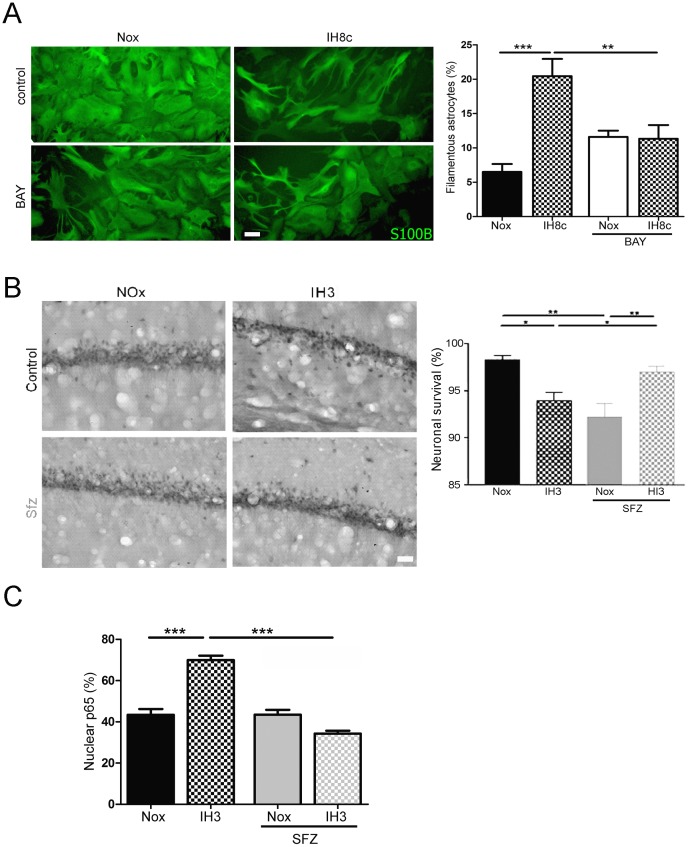
NF-κB is required for astroglial stellation and neuronal alterations induced by IH. A: Astroglial GFAP immunostaining in the dissociated mixed hippocampal cell culture after 8 cycles of IH exposure (IH8c) or normoxia (Nox) treated with the NF-κB chemical blocker BAY117082 (2 µM) or vehicle, bar  = 30 µm. The graph shows the quantitative analysis of the percentage of filamentous astrocytes in a similar experiment. B: NeuN immunostaining in the hippocampal CA-1 region of animals exposed to IH for three days (IH3) and treated with intrahippocampal administrations of sulfasalazine (SFZ), an NF-κB chemical blocker, or vehicle, bar  = 40 µm. The graph shows the quantitative analysis of the neuronal survival based on the morphology of NeuN immunostaining. C: Quantitative analysis of nuclear p65 immunostaining in animals exposed to three days of IH (IH3) which received SFZ or vehicle intrahippocampally. Data on the graphs are shown as means ±SEM; significance vs. control group was represented as indicated: * p<0.05; **p<0.01; *** p<0.001 after two way ANOVA and Bonferroni post-test.

Dissociated mixed hippocampal cell cultures were treated with the NF-κB chemical blocker BAY117082, and then exposed to the IH cycles. As shown in Figure 6A, astrocytic stellation induced by IH exposure was abolished by BAY117082 treatment. However, blockage of NF-κB showed a tendency to increase stellation in normoxic cultures (Figure 6A). Animals which were exposed to IH and received the chemical NF-κB blocker sulfasalazine showed an increase in neuronal survival, compared to those receiving vehicle (Figure 6B). Interestingly, blockage of NF-κB induced a reduction in neuronal survival in normoxic conditions (Figure 6B). The effectiveness of the NF-κB blockage by sulfazalazine was evaluated by studying nuclear localization of NF-κB p65 subunit. Sulfazalazine treatment abolished the increased p65 nuclear localization induced by IH exposure *in vivo* (Figure 6C).

## Discussion

Experimental studies on rodents using IH exposure to mimic human SA are accepted models for analyzing the neurobiological basis of the cognitive alterations observed in human SA patients [13], [48]. These models are supported by the fact that structural alterations have been observed in brains of IH-exposed animals, as well as in diagnostic brain imaging in humans that suffer SA (see for review [10]). Clinical and experimental evidence points towards a predominant role of hippocampus in the cognitive impairments observed in SA patients [10].

The dissection of molecular cascades and cellular events in SA has been quite complex due to the absence of cellular *in vitro* models useful for testing the participation of intracellular mediators in SA. Such models would also be useful for testing neuroprotective drugs before going into animal models. In this work we have used the dissociated mixed hippocampal culture as described by Parpura et al. [49] combined with the IH exposure paradigm described by Shan et al. [36], and demonstrated that eight cycles of IH *in vitro* produce a similar increase in oxidative stress, as determined by TBARS abundance, to that observed in animals exposed to experimental IH.

Over the last years it has been reported that acute necrotic injuries, but also chronic mild injuries to the brain parenchyma, induce the release of DAMP proteins capable of activating innate immunity. Once in the extracellular milieu DAMPs interact with pattern recognition receptors (PRR) like RAGE and receptors of the toll-like family (TLR) to induce different responses, though most of them share the common NF-κB downstream pathway. Following PRR activation, NF-κB transcriptional activity induces the expression of a plethora of genes related to the control of cell survival, but several of them are directly involved in the inflammatory response. While the participation of DAMP/PRR cascade in acute pathological states like ischemia and traumatic brain injury has been addressed, the question of whether these molecules affect neuronal survival in SA remained to be cleared.

RAGE expression has been previously demonstrated to occur in anatomical areas related to the behavioral alterations observed in patients and animal models of SA [11], [22]. On the other hand, members of the S100 family have been recognized as DAMP molecules capable of activating innate immunity [50]. In particular, S100B, which has a glial origin, behaves as a glial-specific DAMP being released after brain injury [51]. In SA patients, S100B levels are increased [32], [33], [52], [53], and we have previously reported that S100B expression dramatically increased in the animal model of SA [11]. Extracellular S100B interacts with RAGE and leads to NF-κB activation [29], [30].

We here used NF-κB reporter mice to demonstrate that this transcription factor is transcriptionally active after IH exposure. In agreement with this observation, the expression of some NF-κB dependent genes (IκB, XIAP, Bcl-XL) is increased in the hippocampus of rats exposed to IH cycles. These results correlate with data showing that the NF-κB activity is increased in circulating monocytes, endothelium, liver, heart and lungs in SA patients [45]–[47], [54] and supports the hypothesis of a pro-inflammatory status as a crucial pathogenic contributor to SA [55]. We observed that increased NF-κB activity is also present in the brain of IH exposed animals and, more importantly, NF-κB activity seems to be related to the neuronal and glial alterations observed after IH exposure. By using dissociated mixed hippocampal cell culture, a setting that contains all the cellular elements (glia and neurons) present in the hippocampus, we observed that NF-κB activity is increased in neurons and glial cells, being the latter probably responsible for the increased pro-inflammatory activity.

Reactive gliosis is a key component of the cellular response to CNS injury and comprises several changes in astrocytes and microglia. In particular, astrocytes suffer the transition from the quiescent to the reactive astrocytic state, accompanied by an increase in intermediate filaments, predominantly GFAP, leading to an increase in soma size and metabolic processes (reviewed in [56]). The beneficial or detrimental effects of reactive gliosis are still a matter of debate, but it is clear enough that the astroglial conversion to the pro-inflammatory phenotype induces neuronal degeneration and death [28].

In order to establish the role of the S100B/RAGE/NF-κB pathway in the reactive gliosis and neuronal alterations observed in the animal model of SA, we performed *in vitro* and *in vivo* loss of function studies on each member of the cascade.

By using S100B blocking antibodies, we observed that neuronal survival after IH was not modified by reducing S100B biological activity. However, reactive gliosis was significantly reduced in the same paradigm both *in vitro* and *in vivo*. This result is in agreement with the idea that S100B is an autocrine factor that may induce a conversion of astrocytes to the reactive phenotype [30], [31]. Our results also showed that S100B blockage in normoxic conditions induces reactive gliosis in absence of injury. This interesting result is not surprising if we consider that S100B is constitutively secreted by astrocytes in the healthy brain, probably acting as an autocrine glial communication molecule. Accordingly, an S100B basal level is expected to occur in the intact CNS, acting as an autocrine system connecting astrocytes; thus, a reduction or suppression of such activity could be interpreted as a stress signal by the astrocytes.

We then studied the next step in the signaling pathway. RAGE blockage with neutralizing antibodies prevented the IH-induced neurite shortening *in vitro* and the reduced neuronal survival *in vivo*. By using the dominant-negative RAGE (RAGEΔcyto) we also found less neuronal loss *in vivo* after IH exposure. Concomitantly, full-length RAGE over-expression reduced neuronal survival. These results indicate that RAGE signaling is necessary to induce neuronal degeneration in this IH paradigm. An increased activity of RAGE-dependent signaling has been related to neuronal degeneration and death, with mechanisms involving reactive oxygen species [57] and NF-κB dependent pro-apoptotic [29], [58] or pro-inflammatory genes [59]. It is tempting to speculate that this latter possibility has a correlation with the fact that we have found in this work that reactive gliosis is blunted by RAGE blockage *in vitro* and *in vivo*. Blockage of RAGE signaling seems to partially prevent astroglial conversion into the pro-inflammatory phenotype, which induces neurodegeneration [28] Intriguingly, S100B or RAGE blockage efficiently reduced reactive gliosis in IH but only RAGE blockage reduced IH-induced neuronal degeneration. This fact points out towards the multiligand capacity of RAGE and the presence of other DAMP able to bind RAGE *in vivo* after IH exposure, being HMGB-1 a candidate for this role. Indeed, HMGB-1 was reported to be increased in the serum of SA patients and was shown to be reduced after CPAP treatment [60]. Although RAGE is not detectable in the adult brain, RAGE blockage *in vivo* was detrimental for neuronal survival and induced reactive gliosis in normoxic conditions. Apparently, a certain level of RAGE basal signaling is required for neuronal survival and/or for preserving astrocytes in the quiescent stage. One hypothesis is that RAGE is undetectable but present in the adult CNS, and that a low basal RAGE signaling is required in the healthy CNS. In fact RAGE mRNA has been detected in the intact brain and blood vessels endothelium [61]. Another possible explanation is that RAGE blockage in circulating macrophages present in the CNS capillaries induces the secretion of cytokines that activate astrocytes and microglia. The requirement of low basal signaling to PRR like RAGE and TLR to maintain certain essential neuronal processes like plasticity is starting to be recognized [62].

RAGE and TLR signaling share the downstream NF-κB dependent transcriptional activity; the last step of the signaling pathway investigated in this work. We found that NF-κB blockage successfully reduced the reactive gliosis induced by IH *in vitro*, and prevented neuronal death induced by IH exposure *in vivo*. NF-κB blockage efficiently reduced p65 NF-κB nuclear localization induced by IH exposure. In normoxia, NF-κB blockage was detrimental and induced reactive gliosis and neuronal degeneration. These results are in agreement with the dual role of NF-κB, by which over-activation induces neuronal death and a pro-inflammatory response in astrocytes and microglia [29]; but certain level of NF-κB activity is required for neuronal survival [34]. An increased NF-κB activity was reported in SA patients in different studies performed on the periphery, including circulating monocytes, endothelium, liver, heart and lungs [45]–[47], [54]. Here we show that NF-κB is also over-activated in the CNS following IH, but more importantly, that either NF-κB blockage or a decreased RAGE signaling reduces neuronal degeneration and reactive gliosis, thus demonstrating that RAGE-NF-κB are involved in the detrimental effects observed after IH exposure as a model of SA.

## Conclusions

Our results demonstrate that activation of the RAGE/NF-κB pathway induces neuronal degeneration, while the S100B/RAGE/NF-κB pathway induces reactive gliosis in the model of SA by IH exposure. Thus, RAGE or NF-κB blockage facilitates neuronal survival and reduces reactive gliosis in the experimental model of SA. However, blockage of these pathways seems to be detrimental in normoxic conditions. As a whole, our work shows that control of PRR activity and NFκB transcriptional events are obvious, but not unique, strategies for the development of neuroprotective interventions to prevent neuronal death in SA.

## Supporting Information

Dataset S1 **Datasets included in figures are available in the file: Datasets.zip.**
(ZIP)
